# Innate Type 2 Responses to Respiratory Syncytial Virus Infection

**DOI:** 10.3390/v12050521

**Published:** 2020-05-08

**Authors:** Allison E. Norlander, R. Stokes Peebles

**Affiliations:** Division of Allergy, Pulmonary, and Critical Care Medicine, Vanderbilt University Medical Center, Nashville, TN 37232-2650, USA; allison.e.norlander.1@vumc.org

**Keywords:** respiratory syncytial virus, ILC2, IL-33, IL-25, HMGB1, TSLP

## Abstract

Respiratory syncytial virus (RSV) is a common and contagious virus that results in acute respiratory tract infections in infants. In many cases, the symptoms of RSV remain mild, however, a subset of individuals develop severe RSV-associated bronchiolitis. As such, RSV is the chief cause of infant hospitalization within the United States. Typically, the immune response to RSV is a type 1 response that involves both the innate and adaptive immune systems. However, type 2 cytokines may also be produced as a result of infection of RSV and there is increasing evidence that children who develop severe RSV-associated bronchiolitis are at a greater risk of developing asthma later in life. This review summarizes the contribution of a newly described cell type, group 2 innate lymphoid cells (ILC2), and epithelial-derived alarmin proteins that activate ILC2, including IL-33, IL-25, thymic stromal lymphopoietin (TSLP), and high mobility group box 1 (HMGB1). ILC2 activation leads to the production of type 2 cytokines and the induction of a type 2 response during RSV infection. Intervening in this innate type 2 inflammatory pathway may have therapeutic implications for severe RSV-induced disease.

## 1. Introduction

Respiratory syncytial virus (RSV) is an important and common cause of infant acute respiratory tract infections and is the primary cause for infant hospitalization in the United States each year [1]. Almost all children are infected with RSV by the age of 2 [2]. Risk factors that predispose infants to severe RSV-associated bronchiolitis include premature birth, young age, immune deficiency, underlying heart or lung disease, as well as neuromuscular disorders [3]. Age at time of infection is another important risk factor for severe RSV-mediated bronchiolitis, with children aged between 2 and 6 months being at the greatest risk for complications and hospitalizations [4]. As lasting immunity to RSV infection is not developed, individuals may experience several RSV infections throughout the course of their lives [5]. In healthy adults, this usually manifests as a mild illness or a ‘cold’ [5]. However, RSV causes severe illness in elderly patients, especially those with preexisting conditions, including individuals who are immunocompromised or those with chronic heart and lung conditions [6,7,8]. Illnesses induced by RSV infection can encompass both the upper and lower respiratory tract [9]. Several days after infection, upper respiratory tract symptoms, such as rhinorrhea and nasal congestion, typically develop. In some individuals, the disease may progress to the lower respiratory tract, resulting in coughing and wheezing, or bronchiolitis, with the severity of disease among infants being quite variable [10]. Severe RSV-induced bronchiolitis results in necrosis and the sloughing of epithelial cells into the airways, airway mucus, edema, and peribronchiolar inflammation, cumulatively resulting in airway obstruction [11]. 

Bronchiolitis and viral pneumonia stemming from RSV infection result in substantial morbidity and mortality in some cases. Current therapeutic options are limited, and treatment is predominantly supportive [12]. There is only one drug, ribavirin, currently FDA approved for the treatment of severe RSV-induced respiratory infections. Ribavirin is a guanosine analog with antiviral properties demonstrated against several viruses, including Zika, hepatitis C, and RSV, making ribavirin a broad-spectrum antiviral [13,14,15]. Ribavirin functions through inhibition of viral replication [16]. However, due to its high cost, lack of specificity, and low ability to control the symptoms, its clinical application is limited [17,18]. Palivizumab is approved by the FDA for immunoprophylaxis for RSV. Palivizumab is a monoclonal antibody that is specific for an epitope located on the F protein [19]. When given prophylactically, palivizumab successfully reduced hospitalizations of children caused by RSV-induced illness [20,21]. Again, due to its high cost, the use of palivizumab clinically is limited and is reserved for use in high-risk populations that include infants that are premature, have low birth weight, have underlying cardiopulmonary diseases, or are immunocompromised [22,23,24]. As a result of the lack of effective post-infection therapeutics, complications from severe RSV infection account for a substantial clinical and economic burden in developed and developing nations alike [25,26,27].

**Figure 1:**
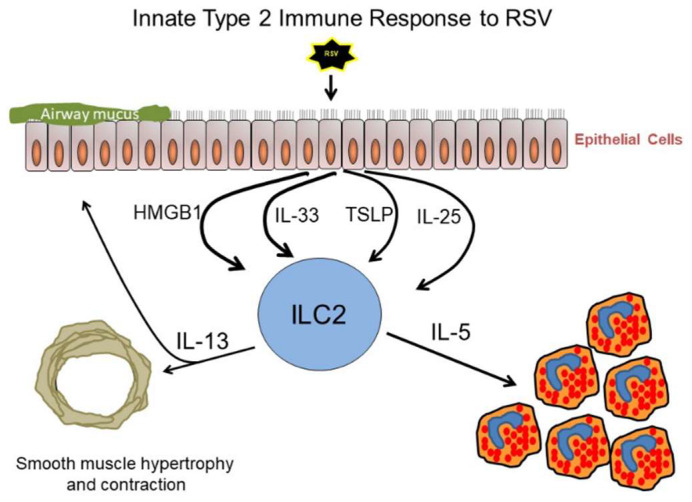
Innate type 2 immune response to respiratory syncytial virus (RSV). RSV infection of airway epithelial cells can result in the release of thymic stromal lymphopoietin (TSLP), interleukin (IL)-33, high mobility group box 1 (HMGB1), and IL-25 by these cells. These alarmin proteins released from RSV-infected epithelial cells can activate group 2 innate lymphoid cells (ILC2) to produce the type 2 cytokines IL-5 and IL-13. IL-5 is the most important eosinophil growth, differentiation, and survival factor. IL-13 has many immunologic and physiologic effects, including promoting airways responsiveness and mucous cell metaplasia.

Both the innate and adaptive immune systems participate in the antiviral response to RSV. The immune response to RSV is generally classified as a type 1 response. Important aspects of the type 1 immune response to RSV include marked production of interferon-γ (IFN-γ) by natural killer (NK) cells and natural killer T (NKT) cells of the innate immune system, and RSV-epitope specific CD8+ T cell mediated viral clearance [28,29,30]. Interestingly, children who developed more severe RSV-mediated bronchiolitis as infants were at elevated risk of developing asthma later in life [31,32,33]. Several factors can influence the severity of RSV infection and the development of RSV-mediated bronchiolitis, one of which includes gender [34]. Gender is also a risk factor for childhood asthma [35]. Male sex is a risk factor for severe RSV illness and boys are twice as likely as girls to develop childhood asthma; to date, bronchiolitis has only been identified as a risk factor for the development of wheeze through adolescence in boys [34,36,37]. Corticosteroids, a therapy often used to mitigate type 2 immune responses in asthma, were ineffective at reducing RSV-induced disease severity and preventing hospitalizations [38,39,40]. Studies of RSV infection in mice reveal that type 2 cytokines, such as interleukin (IL)-4, IL-5, and IL-13, that will be discussed below, may also be expressed during the course of infection. Expression of type 2 cytokines during RSV infection may result in airway mucus and wheezing that are associated with increased disease severity [41,42]. Different strains of RSV have been shown to induce differential immune responses in mice in regard to the types of cytokines the host produces. RSV A2, a strain widely used in in vitro and in vivo mouse experiments, induced an almost exclusive predominant Th1 response in BALB/c mice. However, other clinical isolates of RSV induced greater type 2 cytokine production in BALB/c mice [43,44,45]. Whether such viral strain-specific type 2 immune responses occur in human infection is controversial and is a subject of ongoing investigation. CD4+ Th2 cells are classically associated with the production of the type 2 cytokines IL-4, IL-5, and IL-13. IL-13 signaling promotes goblet cell mucin expression and the development of airway hyperresponsiveness (AHR), or narrowing of airways in response to stimuli [46,47]. IL-5 signaling recruits eosinophils to the lungs and enhances their survival [48]. Eosinophil infiltration during RSV infection is controversial, some studies have found airway eosinophilia, while others have not [49,50]. IL-4 signaling drives Th2 cell differentiation and promotes B cell antibody class switch to IgE [51,52]. However, group 2 innate lymphoid cells (ILC2), a newly described cell type, are also a significant source of IL-13 and other type 2 cytokines in several different pulmonary diseases [53,54,55,56,57,58]. Furthermore, the epithelial-derived cytokines interleukin-33 (IL-33), interleukin-25 (IL-25), and thymic stromal lymphopoietin (TSLP), as well as the innate immune cell-derived cytokine high mobility group box 1 (HMGB1), activate and induce ILC2 function, thereby promoting the progression of type 2-mediated pulmonary diseases (Figure 1) [59,60,61,62,63,64]. Uncovering the contributions of these epithelial-and-innate-derived cytokines and ILC2 to the pathogenesis of severe RSV infection has important implications for treatment. There are currently several biologic therapies that target different mediators of innate type 2 inflammation that are either FDA approved or in clinical trials that could be repurposed for the treatment of RSV-induced bronchiolitis. One such drug is dupilumab, an IL-4 receptor alpha (IL-4rα) antagonist. Both the receptors for IL-4 and IL-13 contain the IL-4rα subunit, thus, blocking this subunit could reduce signaling by both IL-4 and IL-13. Dupilumab is FDA approved for the treatment of chronic rhinosinusitis with nasal polyps, moderate-to-severe asthma, as well as eczema and moderate-to-severe atopic dermatitis in adolescents [65,66,67]. Additionally, an anti-IL33 antibody and an anti-IL33 receptor (ST2) antibody, in separate phase II clinical trials, have recently been evaluated for their ability to treat asthma [68,69,70]. A second anti-IL-33 antibody has completed a phase IIa trial [71,72]. This review will focus on summarizing recently published research highlighting the contributions of ILC2, IL-33, IL-25, HMGB1, and TSLP to RSV pathogenesis. Articles were identified through a PubMed search for key terms (RSV, ILC2, IL-33, IL-25, HMGB1, TSLP) and any combination thereof that was conducted on April 30, 2020. Articles published after 2010 were included in this review.

## 2. Group 2 Innate Lymphoid Cells (ILC2)

ILC2 are a recently described cell type of the innate immune system. These cells do not express rearranged antigen receptors, unlike T and B cells, but are able to produce type 2 cytokines at high levels when activated [73,74]. ILC2 are directly activated by the epithelial-derived cytokines TSLP, IL-33, IL-25, and HMGB1. As such, ILC2 express the corresponding receptors for these cytokines that include TSLPR, ST2, IL-25R, and the receptor for advanced glycation end products (RAGE), as well as toll like receptors (TLR) 2 and 4 [64,73,74,75,76]. ILC2 and the cytokines they produce contribute significantly to the pathology of asthma and allergic diseases [73,74]. Infants hospitalized with severe RSV infection had detectable ILC2 in their nasal aspirates and their frequency was greater than in infants with moderate disease [77]. Moreover, levels of type 2 and epithelial-derived cytokines (IL-33 and TSLP) were significantly higher in the nasal aspirates of infants with severe disease than in those with moderate disease [77]. Furthermore, infants with lower gestational age were confirmed to be at greater risk for severe RSV infection, as were infants with higher levels of IL-4 in their nasal aspirates, when the relationships of individual variables examined in the study and disease severity of the infants were analyzed to identify associations using bivariate logistic regression [77]. These studies provide an important link between ILC2 and RSV-induced bronchiolitis in infants. STAT1 signaling in mice is crucial for restraint of ILC2 responses and prevention of type 2 pathophysiology to RSV infection [78]. Enhanced numbers of ILC2 were present in mice deficient in STAT1 compared to WT mice during the course of RSV infection [78]. Additionally, viral clearance was impaired in STAT1 deficient mice compared to WT mice [78]. Absence of STAT1 signaling increased lung IL-33 expression following RSV infection and abrogation of IL-33 attenuated the enhanced RSV-induced ILC2 response in animals lacking STAT1 signaling [78]. High levels of type I, II, and III interferons are produced during viral infection and all signal through STAT1 [79]. STAT1 signaling is important for the generation of Th1 polarized cells, as well as the production of IFN-γ during viral infection, both of which are essential for recovery and viral clearance [78,80]. Whether STAT1 signaling regulates RSV-induced disease and ILC2 activation in humans is currently unknown. However, a recent study has provided evidence that further warrants the evaluation of STAT1 signaling in the context of severe RSV-mediated infection in humans. The study noted that infants with severe RSV-induced bronchiolitis have reduced levels of type I IFNs and a reduced viral load when compared to children with mild infection [81]. Based on data from mice, this reduction in type I IFN level in infants with severe RSV-induced bronchiolitis likely results in reduced STAT1 activation that favors the generation of type 2 response. RSV infection also increased the expression of OX40L, a marker important for the formation of Th2 cells [82], on ILC2 [83]. Subsequent interactions of OX40L+ ILC2 with OX40 expressing T cells promoted the survival and expansion of Th2 populations [83]. Reciprocally, IL-2 production by CD4+ T cells in the lungs promoted the proliferation of cytokine producing ILC2 during the course of RSV infection [84]. The current evidence suggests that ILC2 activation may promote type 2 immune responses that might worsen RSV-induced disease; however, there are no intervention studies to date that have tested this hypothesis, and such a study would help to elucidate the true role of ILC2 in RSV infection.

## 3. Thymic Stromal Lymphopoietin (TSLP)

TSLP is an IL-7 like cytokine and can influence the development of both T and B cells. TSLP signals through its receptor TSLPR, a heterodimer composed of the IL-7 receptor alpha chain (IL-7rα) and the TSLPR chain [85,86]. TSLP is primarily expressed by epithelial cells; however, keratinocytes, dendritic cells, and mast cells also produce TSLP [87]. TSLP induces cell proliferation, as well as the maturation of dendritic cells (DCs) [88]. TSLP is an important mediator for the development of allergic asthma. Elevated amounts of TSLP were present in the cells within the airway [89] and BALF [90] of patients with asthma and in the lungs of mice with antigen-induced allergic inflammation [91]. Importantly, TSLP levels were upregulated in nasopharyngeal aspirates (NPAs) collected at time of admission of hospitalized infants with viral bronchiolitis, 70% of which were hospitalized due to RSV infection, compared to samples collected from healthy infants at their primary care appointments [92]. Moreover, increased levels of TSLP, as well as of IL-33 and periostin, an extracellular matrix protein downstream of IL-13 [93], were found in the NPAs of patients who were infected with RSV compared to noninfected controls [92]. Identification of TSLP within samples collected from RSV-infected patients demonstrates its potential as a target for mitigation of RSV-induced bronchiolitis in humans. Cultured primary human bronchial epithelial cells produced TSLP in response to RSV infection [94]. Furthermore, cultured primary human bronchial epithelial cells from asthmatic children produced more TSLP in response to RSV infection than cells collected from healthy children [94]. The TSLPR was upregulated on epithelial cells from the human lung epithelial cell line A549 in response to RSV infection [95]. This study also noted that primary human bronchial epithelial cells collected from asthmatic patients had upregulated TSLPR expression in response to RSV infection compared to those collected from healthy patients [95]. Together, these studies demonstrate the expression of TSLP in human-derived cultured cells in response to RSV infection, further demonstrating that RSV infection induces TSLP production. Furthermore, a study in mice has shown that RSV-induced lung IL-13 and airway mucus were blunted in animals that lacked the TSLPR [94]. An important question, however, is whether TSLP is altering immune cell function during RSV infection. This seems to be the case, as co-culture of RSV-infected primary rat epithelial cells (PRAECs) with myeloid dendritic cells (mDCs) resulted in the greater maturation (enhanced expression of CD86 and MHCII) of the mDCs than those that were co-cultured with medium-exposed PRAECs [96]. This response was abrogated when TSLP was knocked down in the epithelial cells with siRNA [96]. Furthermore, mDC expression of OX40L, was upregulated in mDCs co-cultured with RSV-infected PRAECs compared to those co-cultured with medium exposed PRAECs [96]. This response was again abrogated when TSLP was knocked down in the epithelial cells [96]. Administration of an anti-OX40L antibody during primary infection of neonatal mice resulted in reduced AHR, as well as reduced IL-5 and IL-13 levels in the bronchoalveolar lavage fluid (BALF) from mice when they were re-infected compared to mice treated with a control antibody [97]. Similar reductions in AHR and levels of IL-5 and IL-13 in the BALF were seen after re-infection when anti-TSLP was administered prior to the primary infection of neonatal mice compared to mice administered a control antibody [97]. Moreover, administration of an anti-TSLP antibody prior to RSV infection of neonatal mice resulted in a reduction in the number of DCs that accumulated in the lung, as well as their expression of OX40L [97]. These two studies demonstrate an indirect mechanism through which TSLP enhances IL-13 production mediated by OX40L-OX40 interaction between DCs and T cells. Additionally, TSLP, confirmed through antibody neutralization and the use of TSLPR deficient mice, directly increased the number of IL-13 producing ILC2 in response to RSV infection [98]. TSLPR-deficient mice infected with RSV had reduced airway mucus visualized by periodic acid-Schiff (PAS) staining and reduced AHR compared to WT mice infected with RSV [98]. Moreover, the dependency of activation and accumulation of IL-13+ ILC2 on TSLP signaling was demonstrated across several different clinical isolates of RSV and is thus a conserved feature of RSV strains able to induce type 2 immune responses in mice [98]. Sex differences also impact the amount of TSLP present during RSV infection. Neonatal male mice infected with RSV exhibited higher viral loads, as well as lower production of IFNβ, at day 4 post-infection, leading to delayed resolution of infection compared to neonatal female mice infected with RSV [99]. Male mice had also formed bronchus associated lymphoid tissue (BALT) at day 14 post-infection that was not present in female mice [99]. At 4 weeks post-infection, male mice had higher levels of TSLP and IL-33 in the lungs, as well as increased airway mucus visualized by PAS staining and by levels of Muc5ac by quantitative PCR, more lung ILC2, DCs, and OX40L+ cells compared to female mice [99]. These changes in resident lung immune cell populations resulted in male mice becoming more susceptible to allergic exacerbation upon allergen challenge at 4 weeks old compared to female mice [99]. The increased susceptibility to allergic exacerbation at 4 weeks post-RSV infection was reduced in male TSLPR-deficient mice compared to WT males, indicating that TSLP was responsible for inducing the alterations in immune profile seen in male mice [99]. Together, these data suggested an important role for TSLP in the promotion of the type 2 response to RSV infection. 

## 4. Interleukin-33 (IL-33)

IL-33 is an alarmin, or a molecule that when released can signal the presence of tissue or cell damage, and is a member of the interleukin 1 (IL-1] family of cytokines [100]. IL-33 is predominantly expressed and released by endothelial and epithelial cells but can be released or secreted from other cell types within the airway, including macrophages and DCs [100,101,102]. IL-33 binds to and signals through its receptor ST2 (IL-1RL1] [100]. IL-33 can promote type 2 responses [100], and as such, IL-33 is an important mediator of asthma and other allergic diseases. IL-33 can activate and induce ILC2 to produce type 2 cytokines and may activate DCs to promote Th2 polarization [103]. IL-33 can also activate non-type 2 cells that exhibit ST2 expression [104]. Clinically, increased amounts of IL-33 and IL-13 have been found in nasal aspirates of hospitalized infants with RSV, linking IL-33 to type 2 inflammation generation in response to RSV infection [105]. In response to RSV infection, both alveolar macrophages and DCs from the lungs of infected mice became a source of IL-33 as evidenced by flow cytometry of total homogenized lung cells [102]. While assessing lung IL-33 expression by flow cytometry is technically challenging given the long isolation process and resultant loss of cell viability, IL-33 reporter mice have made possible the flow cytometric analysis of IL-33 expression in the setting of RSV infection. For instance, our group recently showed that there was increased lung epithelial cell expression of IL-33 twelve hours after RSV infection. This flow cytometry data correlated with lung protein levels of IL-33 measured by ELISA [106]. Upregulation of IL-33 mRNA in response to RSV infection was confirmed in vitro using the macrophage cell line RAW264.7 and the DC cell line DC2.4 [102]. This increase in IL-33 production was mediated by toll like receptors (TLRs) 3 and 7 [102]. Furthermore, IL-33 produced by macrophages after RSV infection was dependent upon signaling through the MAPK pathway [101]. ILC2 were also activated by IL-33 in response to RSV [107]. Incubation of whole lung cell suspensions from RSV infected mice with an anti-ST2 antibody reduced the number of IL-13+ ILC2 compared to untreated cells [107]. Age at time of infection is an important risk factor for severe RSV-mediated bronchiolitis [4]. IL-33 is produced shortly after birth and results in a wave of ILC2 colonization of the lungs [108]. Activation of ILC2 by endogenous IL-33 during this is important for the development of type 2 immunity [109]. Moreover, there is evidence that demonstrates a link between the production of IL-33 during the perinatal period and the development of asthma [110]. Correspondingly, levels of IL-33 and IL-13 in response to RSV infection differ greatly based on age. Neonatal mice mounted rapid accumulation of IL-13 and IL-33 in the lungs in response to RSV infection that was not seen in adult mice [105]. Higher numbers of lung ILC2 were also seen in neonatal mice and not adults following RSV infection [105]. Interestingly, pretreatment of neonatal mice with an anti-IL-33 antibody prior to RSV infection reduced levels of lung IL-13 and ILC2, and this was not seen in adult mice [105]. Development of AHR was abrogated and numbers of CD4+ Th2 cells were reduced in re-infected neonates that had been pretreated with an anti-IL-33 antibody during primary infection compared to mice that received a control antibody [105]. ST2-deficient mice were protected from the development of type 2 responses when infected with RSV [105]. In one mouse model of RSV infection, AHR was driven by IL-33 activated ILC2 producing IL-13 as a consequence of RSV infection [111]. All of these recent studies provide a clear link between accumulation of IL-33 and induction of an ILC2 mediated type 2 response during RSV infection. However, few studies have uncovered potential therapies to mitigate this response. One such study, identified a glucagon-like peptide 1 receptor (GLP-1R) agonist, an FDA approved treatment for type II diabetes, as a potential therapy. The GLP-1R agonist administered during the course of RSV infection reduced RSV-induced IL-33 upregulation in epithelial cells, as well as type 2 immunopathology [106]. A second study reported increased expression of xanthine oxidase in RSV-infected mice and hospitalized infants [112]. Xanthine oxidase stimulates the inflammasome ultimately resulting in the production of uric acid [112,113]. Treatment of RSV infected transformed human airway epithelial cells or primary mouse airway epithelial cells in culture with a xanthine oxidase inhibitor decreased the expression of IL-33 and TSLP [112]. Additionally, treatment of mice during the course of RSV infection with a xanthine oxidase inhibitor decreased the expression of BALF IL-33 and lung IL-13 and reduced the number of ILC2 present in the lungs [112]. Interestingly, interleukin-1beta (IL-1β) expression was also increased in the BALF during RSV infection and treatment of mice with an IL-1 receptor antagonist (IL1-ra) reduced the expression of BALF IL-33 and lung IL-13, as well as the accumulation of ILC2 that occurred as a result of RSV infection [112]. However, IL-1β has also been shown to decrease IL-33, IL-25 and mucus metaplasia in a mouse model of rhinovirus (RV) infection [114]. These opposing results could be due to differences in the microenvironment that is unique to each pathogen. In a third study, Rhein, a traditional Chinese medicine (lipophilic anthraquinone) found in medicinal herbs that has antiviral activities, inhibited RSV-induced activation of the NLRP3 inflammasome and production of lung IL-33 in mice [115]. 

## 5. High Mobility Group Box 1 (HMGB1)

High mobility group box 1 (HMGB1) is a nonhistone chromatin binding protein that loosely binds DNA [116,117]. HMGB1 performs several crucial functions within the nucleus. HMGB1 stabilizes nucleosome formation and acts as a DNA chaperone to enable DNA replication and repair [116,117]. HMGB1 is also involved in V(D)J recombination and transcription [116,117]. HMGB1 promotes autophagy and cell survival when present in the cytoplasm [117,118]. HMGB1 can be released by DCs, macrophages, and NK cells in response to inflammatory stimuli, including infection, and is also released from necrotic and dead cells [116,117]. Like IL-33, HMGB1 is an alarmin and thereby mediates responses of both the innate and adaptive immune systems when released. HMGB1 signals through RAGE, as well as TLRs 2 and 4 [116,117]. Elevated levels of HMGB1 have been detected in the sputum of human asthmatics [119,120]. Moreover, HMGB1 levels were greater in the NPA of infants hospitalized for severe RSV-induced bronchiolitis [121]. Several mouse studies using different inducible models of asthma demonstrated that anti-HMGB1 antibody therapy reduced IL-4, IL-5, and IL-13, as well as airway mucus compared to control antibody [122] or mice not given antibody [123]. Initial experiments demonstrated increased levels of HMGB1 in lungs of RSV infected mice compared to uninfected controls [124]. HMGB1 expression in the human bronchial epithelial cell line 16HBE was increased following infection with RSV compared to un-infected cells [125]. HMGB1 was increased in the lungs of RSV-infected neonatal rats compared to mock-infected controls [125]. Treatment with glycyrrhizin, an inhibitor of HMGB1, at the time of RSV infection reduced the number of RSV-infected transformed human bronchial epithelial cells [16HBE) and primary normal human bronchial epithelial cells [125]. A separate study confirmed release of HMGB1 from transformed human airway epithelial cells infected with RSV, as well as demonstrated that reactive oxygen species (ROS) generation triggered by RSV infection promoted HMGB1 release from transformed human airway epithelial cells [126]. The study also noted that HMGB1 directly increased the release of cytokines, including IFN-γ, from human primary monocytes [126]. A third study also found that RSV infection induced HMGB1 release from transformed human airway epithelial cells [127]. HMGB1 added to cultures was sufficient to induce proinflammatory cytokine production from primary human monocytes, DCs, and eosinophils [127]. Together these studies demonstrate the capacity of RSV infection to induce HMGB1 release. To date, only one study has examined the effect of HMGB1 on type 2 inflammation in the context of RSV infection. In this study, mice were infected with RSV and were subsequently treated with an HMGB1 antibody on days 14 to 20 post-infection [121]. Anti-HMGB1 therapy significantly reduced the concentrations of IL-4, IL-5, and IL-13 in the BALF at day 21 post-infection compared to a group that was infected and treated with control antibodies [121]. Additionally, GATA3 expression, the number of lung eosinophils, AHR, and cellular infiltration into the lung measured by hematoxylin and eosin (H&E) staining and through BALF cell counts, were decreased at day 21 post-infection in RSV-infected mice who received anti-HMGB1 compared to those that did not [121]. In this model, HMGB1 expression was found to be primarily exist within CC10+ club cells inside the lung [121]. Depletion of CC10+ club cells by injections of naphthalene reduced levels of HMGB1 in the lungs and BALF, as well as levels of type 2 cytokines in the BALF, compared to control animals that were infected with RSV and had received sham injections of PBS at day 21 post RSV infection [121]. This study demonstrates a link between HMGB1 and the severity of RSV infection and identifies a cell type important to its release. Further studies investigating the impact of HMGB1 therapy during RSV infection are warranted. It is worth noting that 3 studies have published links between type 2 inflammation and HMGB1 in animals infected with pneumonia virus of mice (PVM), a murine virus that results in infection similar to that of severe RSV-induced infection in infants. The first study showed that neonatal mice deficient in RAGE manifested a reduced type 1 and type 2 IFN response, blunted peripheral DC (pDC) recruitment, higher levels of HMGB1 and higher amounts of airway smooth muscle mass compared to WT mice in response to PVM infection [128]. Furthermore, reinfection of RAGE deficient mice with PVM 42 days after initial infection resulted in increased AHR and goblet cell hyperplasia compared to WT mice, and these endpoints were reduced with the antibody neutralization of HMGB1 [128]. The second study reported that neonatal mice deficient in interferon-β promotor stimulator-1 (IPS-1) that were PVM-infected and subsequently re-infected with PVM developed severe bronchiolitis marked by release of HMGB1 and IL-33 [129]. The third study demonstrated that pharmacological inhibition of the programmed death response (receptor-interacting serine/threonine-protein kinase 1 (RIPK1) or mixed lineage kinase domain like pseudokinase (MLKL) inhibition) both in vitro and in vivo reduced HMGB1 release and attenuated the development of bronchiolitis and type 2 inflammation in vivo in IRF7-deficient mice, a strain of animals predisposed to developing severe bronchiolitis in response to PVM infection [130]. Pharmacological inhibition of the programmed death response in IRF7-deficient mice also reduced BALF LDH measurements, BALF dsDNA measurements, mucin hypersecretion measured through percent of airway epithelial cells that expressed Muc5ac, eosinophilic infiltration into the BALF and airway smooth muscle remodeling compared to untreated IRF7-deficient mice during PVM infection [130]. Together, these studies suggest that following RSV infection, HMGB1 contributed to the development of the type 2 response during the course of infection.

## 6. Interleukin-25 (IL-25)

IL-25, also known as IL-17E, is a member of the interleukin-17 (IL-17) family of cytokines and binds to its receptor, IL-25R, that is comprised of the IL-17RA and IL-17RB subunits. IL-17RB receptor expression is predominantly on epithelial cells and type 2 cells of the immune system, including Th2 cells and ILC2, and IL-25 is important for successful mounting of type 2 effector responses [131,132,133]. IL-17RB is noticeably upregulated in asthma and allergic disease [134]. Signaling of IL-25 during asthma and allergic diseases leads to eosinophilia, increased airway mucus, AHR, and airway remodeling [134,135]. Many cell types express IL-25 in response to specific inflammatory stimuli including eosinophils, basophils, mast cells, epithelial cells, and Th2 cells [135]. IL-25 also plays a role in RSV-mediated disease. IL-25 is upregulated during the course of RSV infection [136]. Subsequently, neutralization of IL-25 during the course of infection or use of IL-17RB deficient mice reduced airway mucus measured by PAS staining, and accumulation of IL-5 and IL-13 measured in re-stimulated draining lymph node cell supernatants [136]. Furthermore, IL-17RB deficient mice were protected from allergic exacerbations resulting from antigen challenge during RSV infection [136]. A second study also noted an increase of IL-25 in the lungs of mice infected with RSV [30]. Mice treated with anti-ASIALO GM1 to deplete their NK cells and subsequently infected with RSV had increased levels of type 2 cytokines in their lungs, levels of IgE in their serum, and number of mucin-producing epithelial cells in their lungs compared to WT mice [30]. The type 2 response seen in NK cell depleted mice was attenuated by treatment with an anti-IL-25 antibody compared to NK cell depleted mice that received an isotype control antibody, suggesting that the enhanced type 2 response seen in NK cell depleted mice is mediated by IL-25 [30]. Additionally, neonatal PVM-infected mice had increased lung levels of IL-25 at day 14 post-infection compared to un-infected mice [137]. Moreover, neonatal mice infected with PVM and later sensitized and challenged with OVA had increased levels of serum IgE and increased expression of IL-4, IL-5, and IL-13 in CD4 T cells as evaluated by quantitative PCR compared to animals that were not infected and sensitized and challenged with OVA [137]. IL-25 may be important for the enhanced type 2 response found in the PVM-infected and later OVA sensitized/challenged mice in this study, but this was not definitively determined. The aforementioned studies suggest a link between IL-25 expression and the development of type 2 inflammation during RSV infection, however, more studies are needed to truly understand the impact of IL-25 signaling during human RSV infection.

## 7. Conclusions

Our understanding of the role the innate immune system and associated epithelial-derived cytokines in the induction of type 2 programming in response to RSV infection has dramatically increased over the past decade, mainly due to the discovery of ILC2. The data discussed in this review strongly implicate ILC2, IL-33, TSLP, HMGB1, and IL-25 as key early mediators of the type 2 response to RSV. Albeit with some discrepancies, some studies strongly implicate IL-33 [105] as the driver of the type 2 response, while others implicate TSLP [98], HMGB1 [138], and IL-25 [139]. It is possible that strain differences in RSV or differences in age elicit differences in the production of early mediators that then drive the type 2 response. Nevertheless, we still have a long way to go to accurately understand how and why this response is induced in response to RSV infection and its association to severe RSV-induced bronchiolitis with the possible relationship to the later development of childhood asthma. It is important to also consider that children who develop severe RSV-mediated bronchiolitis and childhood asthma may already have baseline alterations in the levels of type 2 cytokines and numbers of cells that mediate type 2 responses prior to infection. If this is in fact the case, then infants that are predisposed to asthma, because of genetic or other risk factors, might be more likely to develop severe RSV infections. The conundrum of whether a predisposition for allergic disease and asthma is a risk factor for severe RSV bronchiolitis, or whether severe RSV bronchiolitis is a risk factor for subsequent asthma and allergic disease, is still being investigated. Regardless of the root cause of severe RSV-mediated bronchiolitis, such as host, virus, and/or environmental factors, it is worth to consider investigating emerging therapies directed at type 2 and epithelial-derived cytokine signaling that have recently been approved, or are in the pipeline for approval, for asthma treatment as therapies through clinical trials. The potential benefit for using agents, such as dupilumab and tezepelumab, as treatments for these patients would be two-fold; it would provide concrete evidence of the contribution of innate type 2 inflammation to disease pathogenesis and might also provide a novel therapeutic target. It is also possible that use of therapies to treat RSV-mediated bronchiolitis that target the early acting alarmins like IL-33 may have no effect as they often will not be administered until after the peak response from these cytokines has been initiated. Yet the amount of evidence related to involvement of IL-33 in the pathogenesis of RSV-mediated bronchiolitis justifies the evaluation of those therapies. For instance, in the mouse model of RSV infection, anti-TSLP antibodies administered either 6 or 36 hours after infection resulted in a significant decrease in IL-13-expressing ILC2 [98]. This result suggests that treating patients after infection with biologics targeting cytokines that stimulate ILC2 may represent a successful strategy. Immune modulation is tricky, given the extensive interplay and interdependency on the part of the immune which occurs during the clearance of infection, or really in response to any illness or disease. However, testing these antibody therapies rigorously in randomized, double-blind, placebo-controlled clinical trials may be something to consider, as they may prove to be new therapies for patients that are highly susceptible to severe infection who stand the greatest risk of sustaining lifelong respiratory complications from RSV.
